# Pyrotinib in the Treatment of Women With HER2-Positive Advanced Breast Cancer: A Multicenter, Prospective, Real-World Study

**DOI:** 10.3389/fonc.2021.699323

**Published:** 2021-07-16

**Authors:** Lili Zhang, Xiaohong Wu, Jun Zhou, Mingzhen Zhu, Hao Yu, Yusong Zhang, Yutian Zhao, Zhengxiang Han, Yujiang Guo, Xiaoqing Guan, Xufen Wang, Hong Xu, Li Sun, Jiaxin Zhang, Min Zhuang, Li Xie, Shiyou Yu, Ping Chen, Jifeng Feng

**Affiliations:** ^1^ Department of Oncology, Jiangsu Cancer Hospital & Jiangsu Institute of Cancer Research & The Affiliated Cancer Hospital of Nanjing Medical University, Nanjing, China; ^2^ Department of Oncology, Affiliated Hospital of Jiangnan University, Wuxi, China; ^3^ Department of Thyroid and Breast Surgery, The First People’s Hospital of Lianyungang, Lianyungang, China; ^4^ Department of Oncology, The Second People’s Hospital of Lianyungang, Lianyungang, China; ^5^ Department of Oncology, Zhenjiang First People’s Hospital, Zhenjiang, China; ^6^ Department of Oncology, The Second Affiliated Hospital of Soochow University, Suzhou, China; ^7^ Department of Radiotherapy and Oncology, Affiliated Hospital of Jiangnan University, Wuxi, China; ^8^ Department of Oncology, The Affiliated Hospital of Xuzhou Medical University, Xuzhou, China; ^9^ Department of General Surgery, Wuxi People’s Hospital, Wuxi, China; ^10^ Department of Breast Surgery, Suqian People’s Hospital of Nanjing Drum-Tower Hospital Group, Suqian, China; ^11^ Department of Breast Surgery, The Third Affiliated Hospital of Soochow University, The First People’s Hospital of Changzhou, Changzhou, China; ^12^ Department of Oncology, The First Affiliated Hospital of Soochow University, Suzhou, China; ^13^ Department of Oncology, Xuzhou Central Hospital, Xuzhou, China; ^14^ Department of Thyroid & Breast Surgery, The Affiliated Hospital of Xuzhou Medical University, Xuzhou, China; ^15^ Department of Oncology, The First People’s Hospital of Lianyungang, Lianyungang, China; ^16^ Department of Oncology, Nanjing Drum Tower Hospital, The Affiliated Hospital of Nanjing University Medical School, Nanjing, China; ^17^ Department of Thyroid & Breast Surgery, Suzhou Municipal Hospital, Affiliated Suzhou Hospital of Nanjing Medical University, Suzhou, China; ^18^ Department of Oncology, Yancheng No.1 People’s Hospital, Yancheng, China

**Keywords:** pyrotinib, breast cancer, human epidermal growth factor receptor 2, brain metastasis, real-world

## Abstract

**Background:**

HER2-positive breast cancer was aggressive, resulting in a poorer prognosis. This multicenter study analyzed the real-world data of women treated with pyrotinib-based therapy, aiming to describe their characteristics, treatment regimens, and to investigate the clinical outcomes.

**Methods:**

A total of 141 patients with HER2-positive breast cancer were enrolled from February 2019 to April 2020. Last follow-up time was February 2021. All patients were treated with pyrotinib-based therapy in 21-day cycles. The primary endpoint was progression-free survival (PFS).

**Results:**

The median PFS (mPFS) for pyrotinib-based therapy was 12.0 months (95%CI 8.1-17.8) in all patients. Among the patients with liver metastases, mPFS was 8.7 months (95%CI, 6.3-15.4) compared to 12.3 months (95%CI, 8.8-23.3) for patients without liver metastases (P=0.172). In addition, patients receiving pyrotinib-based therapy as their >2 lines treatment had a numerically lower mPFS than those receiving pyrotinib-based therapy as their ≤2 lines treatment [8.4 (95%CI, 5.9-15.4) *vs*. 15.1 (95%CI, 9.3-22.9) months, P=0.107]. The mPFS was 12.2 months (95%CI, 7.9-18.8) in patients with previous exposure to trastuzumab and 11.8 months (95%CI, 6.8-22.9) in patients without previous exposure to trastuzumab (P=0.732). Moreover, mPFS in patients receiving regimens with and without capecitabine were 15.1 months (95%CI, 10.0-18.8) and 8.4 months (95%CI, 6.7-22.9), respectively (P=0.070). Furthermore, in patients with brain metastases, estimated 6-month PFS rate was 70.0%, and rate at 12 months was 60.0%. Seventy patients with measurable lesions were evaluable for response. The objective response rate was 38.6% and disease control rate was 85.7%. The most common adverse event was diarrhea (85.0%).

**Conclusion:**

Pyrotinib-based therapy showed promising efficacy in patients with HER2-positive breast cancer and was well tolerated, especially in patients treated with pyrotinib as ≤2 lines treatment and receiving regimens with capecitabine. The results of this real-world study further confirmed the intriguing efficacy of pyrotinib.

## Introduction

Breast cancer is one of the most commonly diagnosed malignancy worldwide. Due to lifestyle changes, the incidence of breast cancer is rising in women (1). With the development of early detection and efficient therapies, mortality from breast cancer has decreased. Nevertheless, breast cancer remains an important cause of death (2, 3). Human epidermal growth factor receptor 2 (HER2)-positive is a key oncogenic driver event, with pathogenesis mainly being the activation of the PI3K/Akt and MAPK pathways (4). Overexpression of HER2 occurs in 15%-20% of breast cancer (5).

HER2-positive breast cancer was aggressive, resulting in a poorer prognosis (6, 7). The anti-HER2 agents, such as trastuzumab, pertuzumab, lapatinib, and trastuzumab emtansine (T-DM1), have dramatically improved the survival in patients with HER2-positive breast cancer (8, 9). However, drug resistance remains a major challenge (10, 11). Thus, the continued development of novel therapies is required.

Pyrotinib is a newly-developed irreversible pan-ErbB receptor tyrosine kinase inhibitor inhibiting HER1, HER2, and HER4 (12). In an open-label phase II study, pyrotinib plus capecitabine had significantly longer progression-free survival (PFS) (18.1 months *vs*. 7.0 months, P<0.001) and higher objective response rate (ORR) (78.5% *vs*. 57.1%, P<0.05) than lapatinib plus capecitabine in patients with HER2-positive breast cancer (13). In addition, a randomized phase III trial (PHOEBE) enrolled 267 patients previously treated with trastuzumab. The results showed that the median PFS (mPFS) was significantly longer in patients receiving pyrotinib plus capecitabine than in those receiving lapatinib plus capecitabine (12.5 months *vs* 6.8 months, P <0.0001), and ORR was higher for patients treated with pyrotinib plus capecitabine than those treated with lapatinib plus capecitabine (67.2% *vs* 51.5%). The most common adverse event was diarrhea which was well manageable (14). Several randomized trials showed the promising efficacy of pyrotinib in HER2-positive breast cancer. However, the efficacy of pyrotinib in patients with different baseline characteristics in the actual clinical practice was rarely reported.

This multicenter study analyzed the real-world data of women treated with pyrotinib-based therapy, aiming to describe their characteristics, treatment regimens, and to investigate the clinical outcomes.

## Patients and Methods

### Study Design and Treatment

This was a multicenter, prospective, real-world study conducted at 15 hospitals in China. Patients with HER2-positive breast cancer were enrolled from February 2019 to April 2020. Last follow-up time was February 2021. All patients were treated with pyrotinib-based therapy in 21-day cycles. Starting dose and combination therapy with chemotherapeutic drugs and/or HER2-targeted agents and/or radiotherapy were determined by physicians’ choice based on previous clinical trials results, general health status and willing of patients, and collected in the electronic case report form (13–15). This study was registered with Chinese Clinical Trial Registry (ChiCTR1900021819).

### Patient Population

Patients were eligible if they were aged ≥18 years and pathologically confirmed HER2-positive advanced breast cancer. HER2 status was evaluated according to the American Society of Clinical Oncology/College of American Pathologists guidelines (16). HER2-positive status was identified when (on observing within an area of tumor that amounts to >10% of contiguous and homogeneous tumor cells) there is evidence of protein overexpression by immunohistochemistry or gene amplification by *in situ* hybridization (HER2 copy number or HER2/CEP17 ratio based on counting at least 20 cells within the area) (16). Patients were excluded if they were pregnant or lactating; had been previously treated with pyrotinib; lost information of treatment; or received less than one cycle of treatment with pyrotinib. No limits on prior therapy were required.

### Study Endpoints

The primary endpoint was PFS, which was defined as the time from the beginning of treatment with pyrotinib to confirmed disease progression or death, whichever came first. The secondary endpoints included ORR (defined as the proportion of patients with a best overall response of complete or partial response), disease control rate (DCR defined as the proportion of patients with a best overall response of complete response, partial response, or stable disease) and overall survival (OS, defined as the time from the beginning of treatment with pyrotinib to death from any cause). Tumor response assessments were conducted in patients with measurable lesions by the investigator according to the Response Evaluation Criteria in Solid Tumors (RECIST), version 1.1. Safety assessments were performed using the Common Terminology Criteria for Adverse Events (CTCAE), version 5.0.

### Statistical Analysis

Patient characteristics, treatment regimens and starting dosages of pyrotinib were summarized as frequency count (percentage) or median (range). Median PFS was estimated by the Kaplan–Meier method, and its 95% confidence interval (CI) was calculated using the Brookmeyer-Crowley method. Median PFS between subgroups (brain metastasis, liver metastasis, the lines of pyrotinib-based therapy, prior exposure to trastuzumab, and regimen with capecitabine) were compared using the log-rank test, and the Cox proportional hazard model was used to analyze the hazard ratio (HR) and 95%CI. Analyses were performed using STATA statistical software version 15.1 or MedCalc version 18.2.1. *P*<0.05 was considered statistically significant.

## Results

### Baseline Characteristics

A total of 141 patients were included from February 2019 to April 2020. Baseline characteristics are showed in **Table 1**. Median age was 52 (range 29–78 years). One hundred and thirty-six patients (96.5%) had Eastern Cooperative Oncology Group performance status score <2. Hormone receptor-positive was present in 56.0% of the patients. The metastatic sites were in brain (14.9%), liver (31.2%), bone (44.0%) and lung (54.6%). Trastuzumab was previously administered in the (neo)adjuvant setting, metastatic setting, and both (neo)adjuvant setting and metastatic setting to 54 (43.5%), 88 (71.0%), and 18 (14.5%) patients, respectively. Patients with primary trastuzumab-resistant breast cancer was 12.1%.

**Table 1:** Patient characteristics at baseline.

Characteristic	Patients (N=141)
Age (years), median (range)	52 (29-78)
ECOG performance status, n (%)	
0-1	136 (96.5)
≥2	5 (3.5)
Hormone receptor status, n (%)	
ER and/or PgR positive	79 (56.0)
ER and PgR negative	61 (43.3)
Unknown	1 (0.7)
Metastatic sites, n (%)	
Brian	21 (14.9)
Liver	44 (31.2)
Bone	62 (44.0)
Lung	77 (54.6)
Previous trastuzumab therapy, n (%)	124 (87.9)
For advanced disease	88 (71.0)
As (neo)adjuvant therapy	54 (43.5)
Both	18 (14.5)
Primary resistance to trastuzumab^a^	15 (12.1)

a Primary resistance to trastuzumab was defined as relapse during or within 12 months after adjuvant trastuzumab or progression within 3 months of trastuzumab treatment for metastatic disease.

ECOG, Eastern Cooperative Oncology Group; ER, estrogen receptor; PgR, progesterone receptor.

### Treatment Administration

One hundred and four (73.8%) patients received pyrotinib-based therapy as the second or further line treatment. One hundred (70.9%) patients initiated pyrotinib treatment at 400 mg, 39 (27.7%) patients started with 320 mg, and 2 (1.4%) patients had a starting dose of 160 mg. The patients received the treatment regimens with capecitabine (55.3%), trastuzumab (17.7%), and endocrine therapy, radiotherapy or antiangiogenic drugs (3.5%). Only 11 (7.8%) patients were treated with pyrotinib monotherapy (**Table 2**).

**Table 2:** Treatment administration.

Pyrotinib treatment	Patients (N=141)
Lines of pyrotinib-based therapy for ABC/MBC, n (%)	
1	37 (26.2)
≥2	104 (73.8)
Starting dose of pyrotinib (mg/day), n (%)	
400	100 (70.9)
320	39 (27.7)
160	2 (1.4)
Regimens, n (%)	
Regimen with capecitabine	78 (55.3)
Pyrotinib + capecitabine	69 (88.5)
Pyrotinib + capecitabine + paclitaxel	1 (1.3)
Pyrotinib + capecitabine + trastuzumab	8 (10.3)
Regimen with trastuzumab	25 (17.7)
Pyrotinib + trastuzumab + chemotherapy	18 (72.0)
Pyrotinib + trastuzumab + pertuzumab + chemotherapy	1 (4.0)
Pyrotinib + trastuzumab	4 (16.0)
Pyrotinib + trastuzumab + endocrine therapy	2 (8.0)
Pyrotinib + chemotherapy other than capecitabine	30 (21.3)
Pyrotinib alone	11 (7.8)
Pyrotinib + endocrine therapy	2 (1.4)
Pyrotinib + radiotherapy	2 (1.4)
Pyrotinib + antiangiogenic drug	1 (0.7)

ABC, advanced breast cancer; MBC, metastatic breast cancer.

### Efficacy

**Figure 1:**
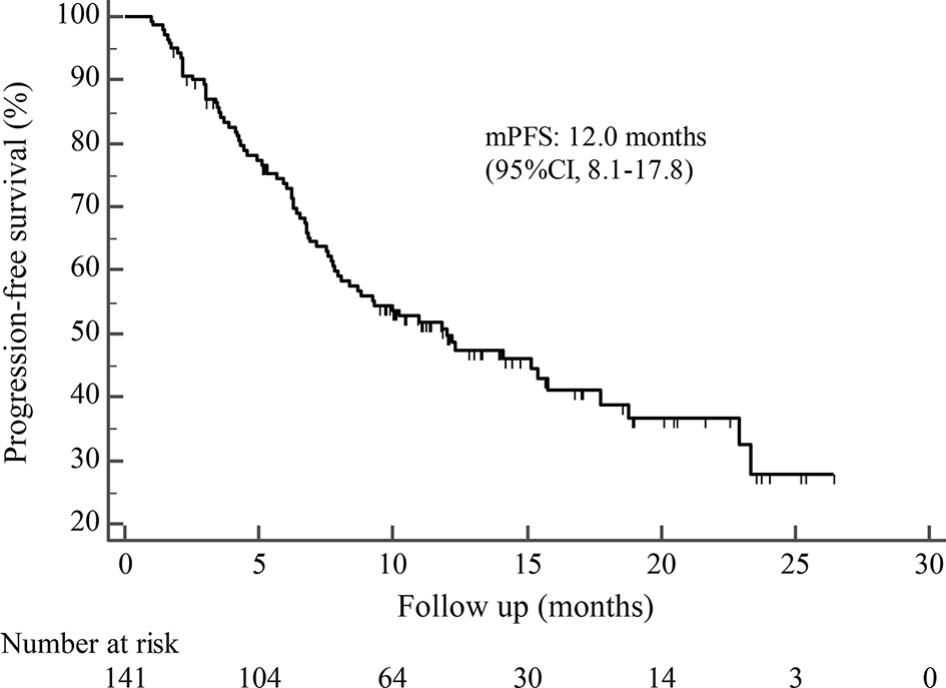
Kaplan-Meier estimates of progression-free survival (PFS) for all patients treated with pyrotinib-based therapy.

**Figure 2:**
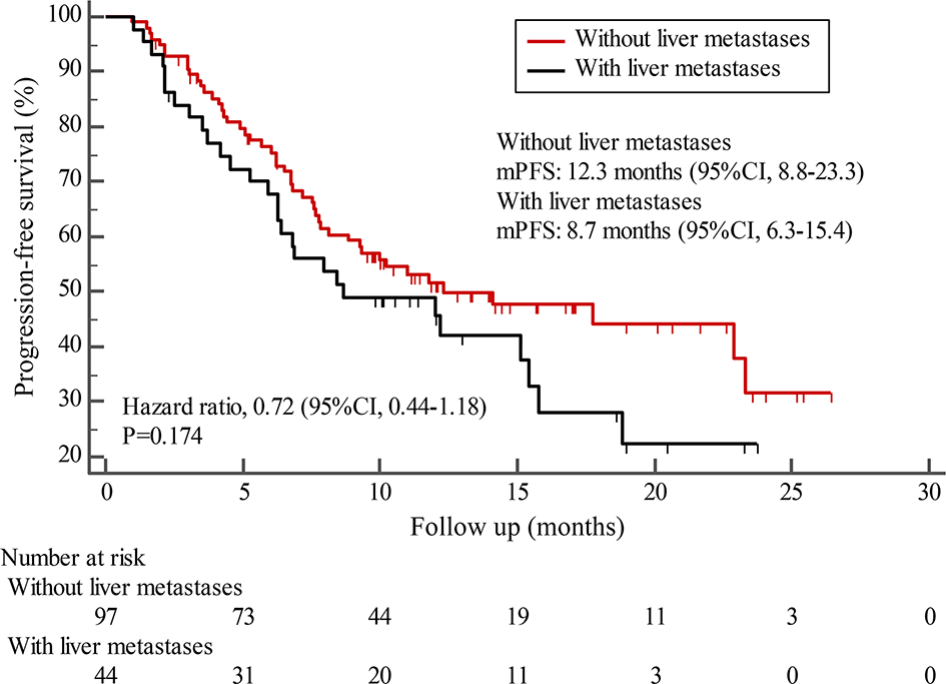
Kaplan-Meier estimates of progression-free survival (PFS) for patients with and without liver metastases.

**Figure 3:**
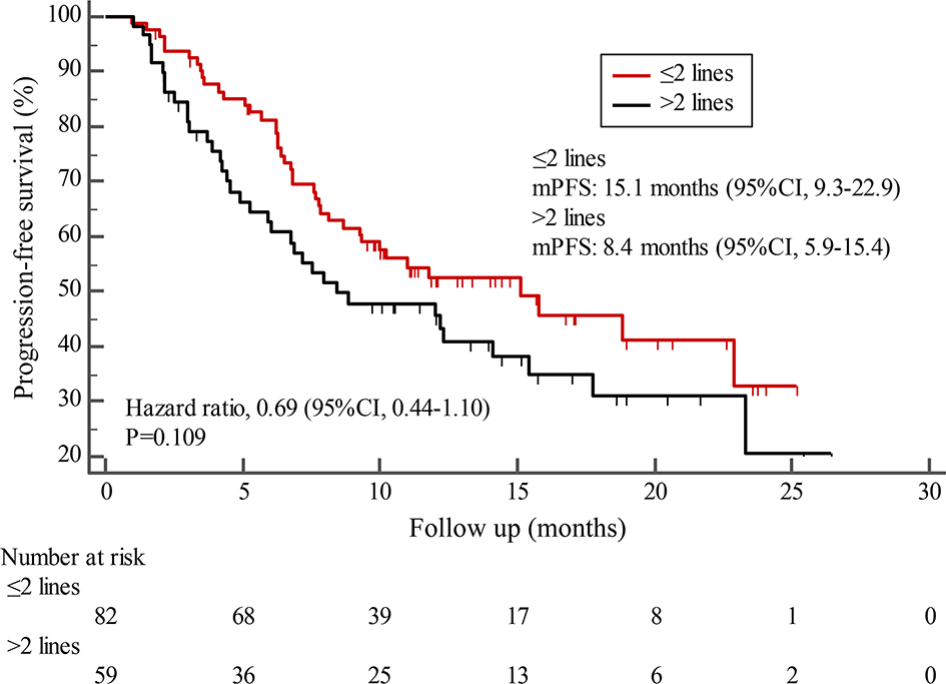
Kaplan-Meier estimates of progression-free survival (PFS) for patients stratified by treatment lines.

**Figure 4:**
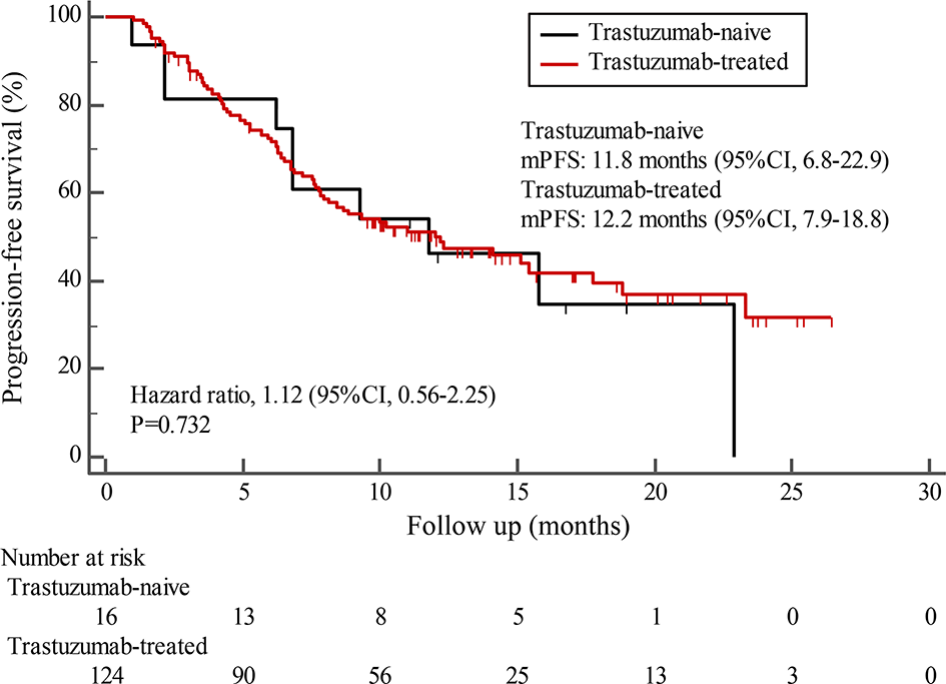
Kaplan-Meier estimates of progression-free survival (PFS) for patients with and without prior trastuzumab exposure.

**Figure 5:**
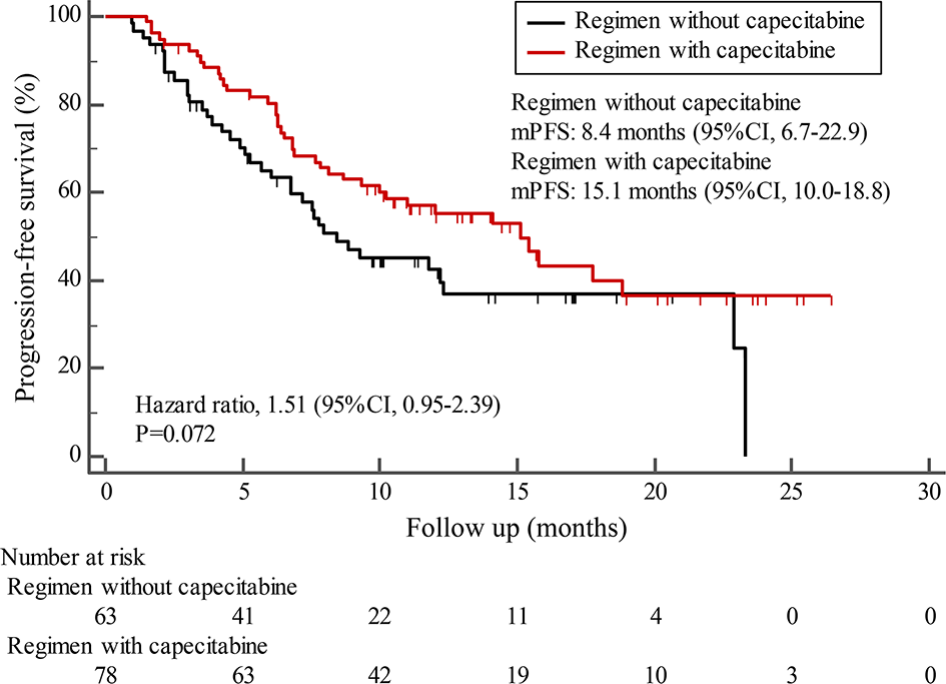
Kaplan-Meier estimates of progression-free survival (PFS) for patients receiving regimen with and without capecitabine.

**Figure 6:**
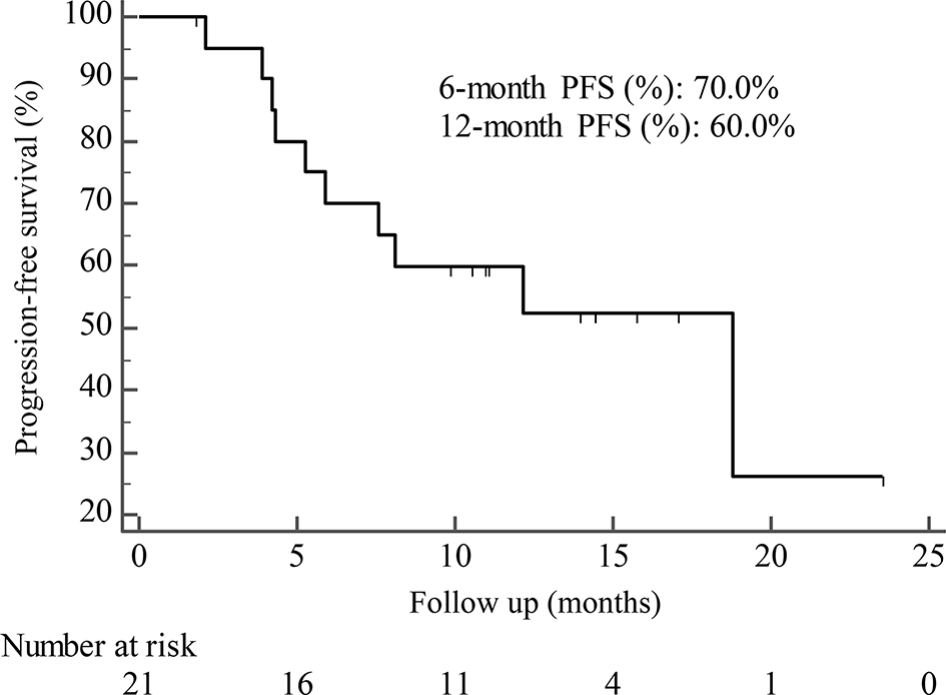
Kaplan-Meier estimates of progression-free survival (PFS) for patients with brain metastases.

By the cutoff date in February 2021, the median duration of follow-up was 11.3 months (range, 1.0-26.4 months) and 52 (45.6%) patients were followed up over 1 year. Fifty-eight (41.1%) patients were still receiving pyrotinib. The median PFS (mPFS) for pyrotinib-based therapy was 12.0 months (95%CI, 8.1-17.8) in all patients (**Figure 1**). Subgroup analyses were conducted according to brain metastasis, liver metastasis, the lines of pyrotinib-based therapy, prior exposure to trastuzumab, and regimen with capecitabine. Among the patients with liver metastases, mPFS was 8.7 months (95%CI, 6.3-15.4) compared to 12.3 months (95%CI, 8.8-23.3) for patients without liver metastases (HR=0.72; 95%CI, 0.44-1.18; P=0.174) (**Figure 2** and **Table 3**). In addition, patients receiving pyrotinib-based therapy as their >2 lines treatment had a numerically lower mPFS than those receiving pyrotinib-based therapy as their ≤2 lines treatment [8.4 (95%CI, 5.9-15.4) *vs*. 15.1 (95%CI, 9.3-22.9) months; HR=0.69; 95%CI, 0.44-1.10; P=0.109] (**Figure 3** and **Table 3**). The mPFS was 12.2 months (95%CI, 7.9-18.8) in patients with previous exposure to trastuzumab and 11.8 months (95%CI, 6.8-22.9) in patients without previous exposure to trastuzumab (HR=1.12; 95%CI, 0.56-2.25; P=0.732) (**Figure 4** and **Table 3**). Moreover, mPFS in patients receiving regimens with and without capecitabine were 15.1 months (95%CI, 10.0-18.8) and 8.4 months (95%CI, 6.7-22.9), respectively (HR=1.51; 95%CI, 0.95-2.39; P=0.072) (**Figure 5** and **Table 3**). Furthermore, in patients with brain metastases, estimated 6-month PFS rate was 70.0%, and PFS rate at 12 months was 60.0% (**Figure 6**). By the data cutoff date, the median OS has not yet been reached.

**Table 3:** Log-rank and Cox multivariate analyses for factors associated with progression-free survival.

Characteristics	Log-rank P	Cox multivariate analysis	
		Hazard ratio (95%CI)	P
Liver metastasis (no *vs*. yes)	0.172	0.72 (0.44-1.18)	0.174
Lines of pyrotinib-based therapy (≤2 *vs*. >2)	0.107	0.69 (0.44-1.10)	0.109
Prior exposure to trastuzumab (no *vs*. yes)	0.732	1.12 (0.56-2.25)	0.732
Regimen with capecitabine (no *vs*. yes)	0.070	1.51 (0.95-2.39)	0.072

Seventy patients with measurable lesions were evaluable for response. The ORR was 38.6% and DCR was 85.7%. One (1.4%) patient achieved complete response and 26 (37.1%) patients had partial response. Thirty-three (47.1%) patients achieved stable disease and 10 (14.3%) patients had progressive disease (**Table 4**). Subgroup analyses of ORR and DCR based on brain metastasis, liver metastasis, the lines of pyrotinib-based therapy, prior exposure to trastuzumab, and regimen with capecitabine are shown in **Table 5**.

**Table 4:** Tumor response in 70 patients with measurable lesions at baseline.

Response	Patients (N=70)
Best response, n (%)	
Complete response	1 (1.4)
Partial response	26 (37.1)
Stable disease	33 (47.1)
Progressive disease	10 (14.3)
Objective response rate, n (%)	27 (38.6)
Disease control rate, n (%)	60 (85.7)

**Table 5:** The subgroup analyses of ORR and DCR.

Best response	ORR (%)	DCR (%)
Brain metastasis		
Yes	45.5	90.9
No	37.3	84.7
P	0.609	0.592
Liver metastasis		
Yes	39.1	87.0
No	38.3	85.1
P	0.946	0.835
Lines of pyrotinib-based therapy		
>2	32.4	79.4
≤2	44.4	91.7
P	0.299	0.143
Prior exposure to trastuzumab		
Yes	36.1	85.2
No	50.0	87.5
P	0.334	0.906
Regimen with capecitabine		
Yes	35.3	85.3
No	33.3	86.7
P	0.663	0.922

ORR, objective response rate; DCR, disease control rate.

### Safety

There were 14 patients without available safety data, leaving 127 patients included in the safety assessments. The adverse events (AEs) of all grades and grade ≥3 were reported in 123 patients (96.9%) and 14 patients (11.0%), respectively (**Table 6**). The most common AE was diarrhea (85.0%), but only 6 patients (4.7%) reported grade ≥3 diarrhea. Moreover, the AEs of all grades that were documented in ≥15% of patients included anemia (37.0%), leukopenia (24.4%), vomiting (24.4%), neutropenia (22.0%), and hyperbilirubinemia (17.3%). No treatment-related deaths were reported.

**Table 6:** Adverse events.

Adverse event, n (%)	Patients (N=127)	
	Any grade	Grade ≥3
Any event	123 (96.9)	14 (11.0)
Diarrhea	108 (85.0)	6 (4.7)
Anemia	47 (37.0)	0
Leukopenia	31 (24.4)	0
Vomiting	31 (24.4)	1 (0.8)
Neutropenia	28 (22.0)	3 (2.4)
Hyperbilirubinemia	22 (17.3)	4 (3.1)
Aspartate aminotransferase increased	15 (11.8)	1 (0.8)
Alkaline phosphatase increased	15 (11.8)	1 (0.8)
γ-glutamyltransferase increased	14 (11.0)	2 (1.6)
Thrombocytopenia	12 (9.4)	0
Rash	12 (9.4)	2 (1.6)
Hypoalbuminemia	12 (9.4)	1 (0.8)
Alanine aminotransferase increased	12 (9.4)	0
Nausea	10 (7.9)	0
Asthenia	7 (5.5)	0
Stomatitis	6 (4.7)	0
Loss of appetite	6 (4.7)	0
Hand-foot syndrome	2 (1.6)	0
Paronychia	2 (1.6)	0
Kidney function abnormalities	2 (1.6)	0
Abdominal pain	1 (0.8)	0
Palpitation	1 (0.8)	0
Dizziness	1 (0.8)	0
Pruritus	1 (0.8)	0

## Discussion

HER2-positive breast cancer is a more aggressive phenotype (17). The anti-HER2 agents (trastuzumab, pertuzumab, lapatinib, and T-DM1) have dramatically improved the prognosis in patients with HER2-positive breast cancer (18). However, primary or acquired resistance to anti-HER2 agents remains a major challenge (10). Thus, the novel therapy is required to provide option to patients.

This study was carried out to analyze the efficacy and safety of pyrotinib-based therapy in patients with HER2-positive breast cancer in the real world. The most inspiring result of the study was a mPFS of 12.0 months, higher than that of trastuzumab (10.9 months) and T-DM1 (10.0 months) in the real-world setting (19, 20), and close to the mPFS result (12.5 months) of pyrotinib in the phase III PHOEBE study (14). In addition, the ORR of pyrotinib-based therapy in this study was 38.6% was also superior to that of T-DM1 (20.0%) (15).

Subgroup results confirmed that the mPFS in patients with liver metastases was 8.7 months, which was shorter than the 12.3 months found in patients without liver metastases. This result is concordant with previous report in which liver metastases correlated with worse survival in patients with breast cancer (21, 22). Moreover, the mPFS was 15.1 months in patients receiving pyrotinib as ≤2 lines treatment, similar to the result of trastuzumab as first-line treatment reported in the phase III PUFFIN study (14.5 months) (23). Our study demonstrated the efficacy of pyrotinib, providing evidence for the patients treated with pyrotinib as ≤2 lines treatment. In addition, 87.9% of patients in our study received prior trastuzumab treatment, thus we assessed the benefit of pyrotinib-based therapy in patients progressed on trastuzumab. The results showed that patients could benefit from pyrotinib, regardless of whether they had been previously exposed to trastuzumab or not. Our results also confirmed that pyrotinib-based therapy with capecitabine achieved a numerically higher ORR and longer mPFS than that without capecitabine, which merits further assessment in the future. In addition, among patients with brain metastases, the 6-month and 12-month PFS rates were 70.0% and 60.0%, respectively, numerically better than that of anti-HER2 monoclonal antibodies in patients with brain metastases (19, 24, 25), indicating that pyrotinib is an important treatment option for patients with brain metastases.

Diarrhea was the most common AE (85.0%), but only 4.7% of patients reported grade ≥3 diarrhea which could be well controlled. The incidence of diarrhea in our study was lower than that in previously studies of pyrotinib (14, 26), which may be the result of the relatively low dose of pyrotinib (only 70.92% of patients were treated with pyrotinib at a starting dose of 400 mg/day). The antidiarrhea treatment or dose reduction after diarrhea could well control the occurrence of diarrhea.

This study confirmed the advantages of pyrotinib. However, the data were acquired from an observational study which included potential information bias or incomplete data. The sample size was small, and the safety profile of 14 (9.9%) patients could not be evaluated due to the missing data. In addition, the median OS has not yet been reached, but the follow up is ongoing. Despite these limitations, the results of this study provide evidence for the real-world use of pyrotinib in patients with HER2-positive breast cancer.

In conclusion, pyrotinib-based therapy showed promising efficacy in patients with HER2-positive breast cancer and was well tolerated, especially in patients treated with pyrotinib as ≤2 lines treatment and receiving regimens with capecitabine. The results of the real-world study further confirmed the intriguing efficacy of pyrotinib.

## Data Availability Statement

The raw data supporting the conclusions of this article will be made available by the authors, without undue reservation.

## Ethics Statement

The studies involving human participants were reviewed and approved by the Ethics Committee of Jiangsu Cancer Hospital. The patients/participants provided their written informed consent to participate in this study.

## Author Contributions

JF and LZ were involved in study conception and design. LZ, XHW, JZ, MZZ, HY, YSZ, YTZ, ZH, YG, XG, XFW, HX, LS, JXZ, MZ, LX, SY, PC, and JF were involved in the acquisition of data. LZ was involved in the analysis and interpretation of data. XHW was involved in drafting the manuscript. LZ was involved in revising the manuscript. All authors contributed to the article and approved the submitted version.

## Funding

This work was supported by the Chinese Society of Clinical Oncology-Hengrui Oncology research foundation (grant number: Y-HR2019-0348). The funding source had no role in the study design, collection, analysis and interpretation of data, writing of the manuscript, or decision to submit the article for publication.

## Conflict of Interest

The authors declare that the research was conducted in the absence of any commercial or financial relationships that could be construed as a potential conflict of interest.

## References

[1] Harbeck N Gnant M. Breast Cancer. Lancet (2017) 389(10074):1134–50. 10.1016/S0140-6736(16)31891-8 27865536

[2] McDonald ES Clark AS Tchou J Zhang P Freedman GM. Clinical Diagnosis and Management of Breast Cancer. J Nucl Med (2016) 57 Suppl 1:9S–16S. 10.2967/jnumed.115.157834 26834110

[3] Abderrahman B Jordan VC. Successful Targeted Therapies for Breast Cancer: The Worcester Foundation and Future Opportunities in Women’s Health. Endocrinology (2018) 159(8):2980–90. 10.1210/en.2018-00263 PMC6963694 29931061

[4] D’Amato V Raimondo L Formisano L Giuliano M De Placido S Rosa R. Mechanisms of Lapatinib Resistance in HER2-driven Breast Cancer. Cancer Treat Rev (2015) 41(10):877–83. 10.1016/j.ctrv.2015.08.001 26276735

[5] Slamon DJ Godolphin W Jones LA Holt JA Wong SG Keith DE. Studies of the HER-2/neu Proto-Oncogene in Human Breast and Ovarian Cancer. Science (1989) 244(4905):707–12. 10.1126/science.2470152 2470152

[6] Loibl S Gianni L. HER2-Positive Breast Cancer. Lancet (2017) 389(10087):2415–29. 10.1016/S0140-6736(16)32417-5 27939064

[7] Waks AG Winer EP. Breast Cancer Treatment: A Review. JAMA (2019) 321(3):288–300. 10.1001/jama.2018.19323 30667505

[8] Swain SM Baselga J Kim S-B Ro J Semiglazov V Campone M. Pertuzumab, Trastuzumab, and Docetaxel in HER2-positive Metastatic Breast Cancer. N Engl J Med (2015) 372(8):724–34. 10.1056/NEJMoa1413513 PMC5584549 25693012

[9] Diéras V Miles D Verma S Pegram M Welslau M Baselga J. Trastuzumab Emtansine Versus Capecitabine Plus Lapatinib in Patients With Previously Treated HER2-positive Advanced Breast Cancer (EMILIA): A Descriptive Analysis of Final Overall Survival Results From a Randomised, Open-Label, Phase 3 Trial. Lancet Oncol (2017) 18(6):732–42. 10.1016/S1470-2045(17)30312-1 PMC5531181 28526536

[10] Vernieri C Milano M Brambilla M Mennitto A Maggi C Cona MS. Resistance Mechanisms to anti-HER2 Therapies in HER2-positive Breast Cancer: Current Knowledge, New Research Directions and Therapeutic Perspectives. Crit Rev Oncol Hematol (2019) 139:53–66. 10.1016/j.critrevonc.2019.05.001 31112882

[11] Sung M Tan X Lu B Golas J Hosselet C Wang F. Caveolae-Mediated Endocytosis as a Novel Mechanism of Resistance to Trastuzumab Emtansine (T-Dm1). Mol Cancer Ther (2018) 17(1):243–53. 10.1158/1535-7163.MCT-17-0403 29054985

[12] Li X Yang C Wan H Zhang G Feng J Zhang L. Discovery and Development of Pyrotinib: A Novel Irreversible EGFR/HER2 Dual Tyrosine Kinase Inhibitor With Favorable Safety Profiles for the Treatment of Breast Cancer. Eur J Pharm Sci (2017) 110:51–61. 10.1016/j.ejps.2017.01.021 28115222

[13] Ma F Ouyang Q Li W Jiang Z Tong Z Liu Y. Pyrotinib or Lapatinib Combined With Capecitabine in HER2-Positive Metastatic Breast Cancer With Prior Taxanes, Anthracyclines, and/or Trastuzumab: A Randomized, Phase Ii Study. J Clin Oncol (2019) 37(29):2610–9. 10.1200/JCO.19.00108 31430226

[14] Xu B Yan M Ma F Hu X Feng J Ouyang Q. Pyrotinib Plus Capecitabine Versus Lapatinib Plus Capecitabine for the Treatment of HER2-positive Metastatic Breast Cancer (PHOEBE): A Multicentre, Open-Label, Randomised, Controlled, Phase 3 Trial. Lancet Oncol (2021) 22(3):351–60. 10.1016/S1470-2045(20)30702-6 33581774

[15] Li F Xu F Li J Wang T Bian L Zhang S. Pyrotinib Versus Trastuzumab Emtansine for HER2-positive Metastatic Breast Cancer After Previous Trastuzumab and Lapatinib Treatment: A Real-World Study. Ann Transl Med (2021) 9(2):103. 10.21037/atm-20-4054 33569405 PMC7867920

[16] Wolff AC Hammond MEH Hicks DG Dowsett M McShane LM Allison KH. Recommendations for Human Epidermal Growth Factor Receptor 2 Testing in Breast Cancer: American Society of Clinical Oncology/College of American Pathologists Clinical Practice Guideline Update. J Clin Oncol (2013) 31(31):3997–4013. 10.1200/JCO.2013.50.9984 24101045

[17] Slamon DJ Clark GM Wong SG Levin WJ Ullrich A McGuire WL. Human Breast Cancer: Correlation of Relapse and Survival With Amplification of the HER-2/neu Oncogene. Science (1987) 235(4785):177–82. 10.1126/science.3798106 3798106

[18] Cesca MG Vian L Cristóvão-Ferreira S Pondé N de Azambuja E. HER2-Positive Advanced Breast Cancer Treatment in 2020. Cancer Treat Rev (2020) 88:102033. 10.1016/j.ctrv.2020.102033 32534233

[19] Hardy-Werbin M Quiroga V Cirauqui B Romeo M Felip E Teruel I. Real-World Data on T-DM1 Efficacy - Results of a Single-Center Retrospective Study of HER2-positive Breast Cancer Patients. Sci Rep (2019) 9(1):12760. 10.1038/s41598-019-49251-5 31484985 PMC6726763

[20] Li Y Gong C Lu Q Zhou Z Luo T Li W. Real-World Data of Triplet Combination of Trastuzumab, Lapatinib, and Chemotherapy in HER2-Positive Metastatic Breast Cancer: A Multicenter Retrospective Study. Front Oncol (2020) 10:271. 10.3389/fonc.2020.00271 32195186 PMC7062863

[21] Cameron D Casey M Oliva C Newstat B Imwalle B Geyer CE. Lapatinib Plus Capecitabine in Women With HER-2-positive Advanced Breast Cancer: Final Survival Analysis of a Phase III Randomized Trial. Oncologist (2010) 15(9):924–34. 10.1634/theoncologist.2009-0181 PMC3228041 20736298

[22] Göksu SS Bozcuk H Koral L Çakar B Gündüz S Tatlı AM. Factors Predicting Lapatinib Efficacy in HER-2+ Metastatic Breast Carcinoma: Does it Work Better in Different Histologic Subtypes? Indian J Cancer (2015) 52(4):517–9. 10.4103/0019-509X.178382 26960462

[23] Xu B Li W Zhang Q Shao Z Li Q Wang X. Pertuzumab, Trastuzumab, and Docetaxel for Chinese Patients With Previously Untreated HER2-positive Locally Recurrent or Metastatic Breast Cancer (PUFFIN): A Phase III, Randomized, Double-Blind, Placebo-Controlled Study. Breast Cancer Res Treat (2020) 182(3):689–97. 10.1007/s10549-020-05728-w PMC7320929 32564260

[24] Krop IE Lin NU Blackwell K Guardino E Huober J Lu M. Trastuzumab Emtansine (T-DM1) Versus Lapatinib Plus Capecitabine in Patients With HER2-positive Metastatic Breast Cancer and Central Nervous System Metastases: A Retrospective, Exploratory Analysis in EMILIA. Ann Oncol (2015) 26(1):113–9. 10.1093/annonc/mdu486 PMC4679405 25355722

[25] Lin NU Borges V Anders C Murthy RK Paplomata E Hamilton E. Intracranial Efficacy and Survival With Tucatinib Plus Trastuzumab and Capecitabine for Previously Treated HER2-Positive Breast Cancer With Brain Metastases in the HER2CLIMB Trial. J Clin Oncol (2020) 38(23):2610–9. 10.1200/JCO.20.00775 PMC7403000 32468955

[26] Ma F Li Q Chen S Zhu W Fan Y Wang J. Phase I Study and Biomarker Analysis of Pyrotinib, a Novel Irreversible Pan-ErbB Receptor Tyrosine Kinase Inhibitor, in Patients With Human Epidermal Growth Factor Receptor 2-Positive Metastatic Breast Cancer. J Clin Oncol (2017) 35(27):3105–12. 10.1200/JCO.2016.69.6179 28498781

